# Fragmented QRS complex frequency and location as predictor of cardiogenic shock and mortality following acute coronary syndrome

**DOI:** 10.1186/s43044-020-00076-y

**Published:** 2020-07-23

**Authors:** Ahmad Salah Younis, Moataz Ibrahim El-Halag, Mahmoud Ali ElBadry, Nora Ismail Mohamed Abbas

**Affiliations:** Department of Critical Care Medicine, Faculty of Medicine, Cairo University, Kasr alAiny Street, Cairo, 11562 Egypt

## Abstract

**Background:**

Worldwide, coronary heart disease (CHD) is topping the foremost important chief causes of mortality. Fragmented QRS (f-QRS) is a pattern of QRS complex in 12 leads surface ECG which showed a promising value in predicting the outcome in cardiac diseases including ischemic heart disease.

We aimed to research the importance of using f-QRS as a non-invasive and cheap tool for the prediction of cardiogenic shock and mortality in acute coronary syndrome (ACS).

**Methods:**

A retrospective study includes eighty four critically ill ACS patients. Patients were classified consistent with the presence or absence of fragmented QRS into two groups (46 and 38 patients respectively). Exclusion criteria include past history of important ischemic events (MI, PCI, and CABG), permanent AF, and/or cardiomyopathy.

No statistical significant differences were detected between the 2 groups as regards the age, gender, major risk factors of ischemic heart condition, cardiac bio-markers, Killip class, LVEF, updated GRACE risk score of ACS, and in-hospital mortality.

**Results:**

A number value of f-QRS leads > 3 yields sensitivity and specificity (83.3% and 72.5% respectively) for predicting hospital mortality. The f-QRS group was further split-up according to the numbers of f-QRS leads into 2 subgroups; subgroup (A1) including patients with > 3 f-QRS leads and subgroup (A2) including patients ≤ 3 f-QRS leads. Subgroup (A2) showed considerable difference as regards some important variables including a higher SBP (*P* = 0.016), a slower HR (*P* = 0.014), a lower up-dated GRACE risk score (3.22 ± 6.95 vs 6.81 ± 12, *P* value 0.048), and a lower rate of hospital death (1/30 vs. 5/16, *P* = 0.015). Anterior f-QRS showed statistically significant higher HR, lower SBP, a higher frequency of shock, a higher updated GRACE risk score, and a higher chance of in-hospital mortality (*P* = 0.004) compared to non-anterior f-QRS.

**Conclusion:**

The position and number of f-QRS leads provide a non-invasive and a readily accessible tool to predict the prognosis, occurrence of cardiogenic shock, and in-hospital mortality.

## Background

Consistent with [16], ischemic heart diseases (IHD) is a major explanation of death and disability in developed countries. It is accountable for about one-third or more of all mortality in individuals over age 35.

Flowers and associates since 1960s [9] were the primary researcher to examine the slurring and morphological changes in QRS complex and documenting the presence of f-QRS complexes fragmented QRS (f-QRS) as defined by [5], is that the occurrence of an additional (extra) R wave (R′) or notching within the nadir of the S wave, or the existence of > 1 R′ (fragmentation) in 2 contiguous ECG leads, regarding a significant coronary artery territory. Moreover, fragmentation in wide complex QRS, i.e., ≥ 120 ms (BBB and paced rhythms) was defined in [6] by Das and associates as various RSR′ patterns with or without a Q wave, with > 2 R waves (R′) or > 2 notches within the R wave, or > 2 notches within the down-stroke or upstroke of the S wave, in 2 contiguous leads regarding a significant coronary artery territory in fragmented BBB and also the presence of > 2 R′ or > 2 notches within the S waves in 2 contiguous leads in fragmented paced QRS. Torigoe and colleagues concluded that the quantity of leads with f-QRS, especially the presence of ≥ 3 leads with f-QRS, is an independent predictor of cardiac death or hospitalization for heart failure in 170 patients with prior MI.

[7] studied the usefulness of fragmented QRS on a 12-lead electrocardiogram. Serial electrocardiograms from 896 patients with ACS (104 with STEMI, 337 with NSTEMI, and 455 with UA) were obtained every 6 to 8 h during the initial 24 h after the diagnosis of MI and also the following day (< 48 h). Fragmented QRS developed in 224 patients (51%) in the MI group and only 17 (3.7%) in the UA group. Kaplan-Meier survival analysis revealed that patients with f-QRS had significantly decreased time to death compared to those without f-QRS. Additionally, the presence of f-QRS at the 48th hour could be a significant predictor of major adverse cardiac events (MACE) in STEMI patients who have undergone primary PCI consistent with [2].

Our aim was to research the worth of using f-QRS as a non-invasive and cheap tool for the prediction of cardiogenic shock, prognosis, and mortality in acute coronary syndrome (ACS).

## Methods

From our medical records of patients admitted between November 2013 and April 2015, this retrospective study was conducted on 84 patients (67 males and 17 females) aging between 29 and 80 years admitted to the Critical Care Department with acute coronary syndrome (ACS). They were divided by 12 leads surface ECG within 2 days of admission into two groups; group A that features 46 patients with f-QRS and group B that has 38 patients without f-QRS. Patients with major ischemic events (prior myocardial infarction, PCI, CABG), permanent AF, and/or myocardial diseases were excluded.

The available medical records of the patients were reviewed for routine medical history, clinical examination including SBP and HR on admission, the risk factors of CHD, routine laboratory measurements including cardiac bio-markers and echocardiography.

**Fig. 1:**
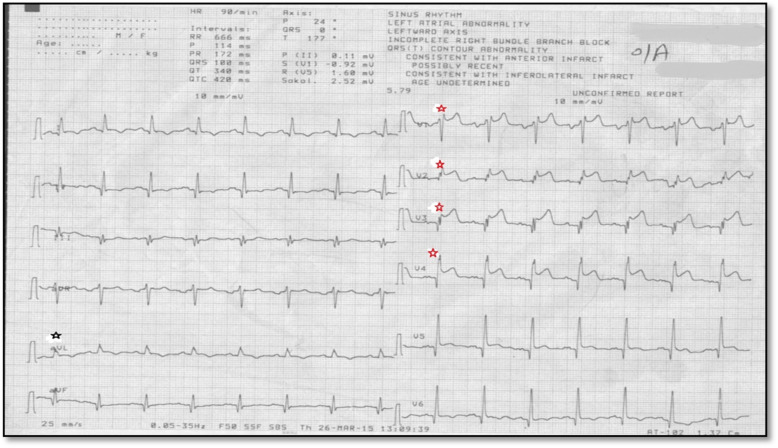
f-QRS in V1-V4 (anterior f-QRS). Note that isolated f-QRS in aVL does not count as lateral f-QRS

ECGs were analyzed for (1) detection of fragmented QRS (f-QRS) within the initial 2 days using diagnostic criteria defined by [5, 6]. (2) Localization of f-QRS: Anterior f-QRS was defined by the presence of f-QRS in 2 contiguous anterior leads (V1 to V5), lateral f-QRS was defined by the presence of f-QRS in 2 contiguous lateral leads (I, aVL, and V6), and inferior f-QRS was defined by the presence of f-QRS in 2 contiguous inferior leads (II, III, and aVF) consistent with [10]. (3) Count the amount of leads with fragmented QRS (at least 2 contiguous leads reminiscent of a significant coronary artery territory) (Fig. 1).

Our outcomes were Killip class, LVEF, calculated updated GRACE risk score, and in-hospital death as a short-term MACE.

Statistical data were described in terms of mean ± standard deviation (± SD), median and range, or frequencies (number of cases) and percentages when appropriate. Comparison of numerical variables between the study groups was done using Student *t* test for independent samples in comparing 2 groups of normally distributed data and/or large groups and Mann Whitney *U* test for independent samples for comparing not-normal data and whenever appropriate. When comparing categorical data, chi-square (*χ*^2^) test was applied. Exact test was performed instead when the expected frequency is fewer than 5. Correlation between different variables was done using Spearman rank correlation equation. Accuracy was represented by means of the terms sensitivity and specificity. Receiver operator characteristic (ROC) analysis was used to determine the optimum cutoff value for number of leads with f-QRS in diagnosing important outcomes. *P* values < 0.05 was considered of statistical significance.

## Results

- Of the 84 patients data analyzed, 46 patients had f-QRS (group A) while 38 patients did not have (group B). The study showed no significant differences between both groups as regards general demographic characteristics, the major risk factors of CHD including HTN, DM, dyslipidemia, and smoking or SBP and HR on admission. Also, in terms of abnormal cardiac bio-markers, Killip class, updated GRACE risk score, and in-hospital death, we could not find any significant difference between the cases and controls group (Table 1).

- Eighteen patients (39% of group A) had f-QRS in 2 leads, 12 patients (26% of group A) had f-QRS in 3 leads while the rest of patients in this group had f-QRS in 4 to 8 leads.

**Table 1:** General demographic characteristic, major risk factors, forms of ACS, (SBP and HR) on admission, positive cardiac biomarkers, location of STEMI, Killip class, risk stratifications

	f-QRS	Non f-QRS	***P*** value
**Age,** years	52.52 ± 10.04	54.29 ± 12.8	*0.480*
**Sex,** m/f	40/6	27/11	*0.071*
**Body weight,** kg	80.22 ± 11.78	79.42 ± 8.94	*0.736*
**Smoking**	36/46	24/38	*0.127*
**Dyslipidemia**	14/35	16/33	*0481*
**HTN**	18/46	16/38	*0.782*
**DM**	16/46	11/38	*0.569*
**STEMI**	38/46	27/38	*0.208*
**NSTEMI**	4/46	7/38	*0.212*
**UA**	4/46	4/38	*1.000*
**SBP,** mmHg	129.78 ± 37.019	129.46 ± 34.717	*0.968*
**HR,** bpm	85.3 ± 17.453	78.18 ± 19.154	*0.079*
**Abnormal cardiac biomarkers**	41/46	32/38	*0.534*
**STEMI in AS zone**	18/38	12/25	*0.961*
**STEMI in IL zone**	20/38	13/25	*0.961*
**Killip I**	33/46	21/38	*0.117*
**Killip II**	9/46	13/38	*0.129*
**Killip III**	0/46	1/38	*0.452*
**Killip IV**	4/46	3/38	*1.000*
**Updated GRACE score**	4.42 ± 8.97	4.24 ± 6.56	*0.8999*
**High risk GRACE**	13/45	15/37	*0.268*
**Intermediate risk GRACE**	22/45	12/37	*0.132*
**Low risk GRACE**	10/45	10/37	*0.614*

*AS* antroseptal; *IL* infrolateral; *DM* diabetes mellitus; *HR* heart rate; *HTN* hypertension; *IL* inferolateral; *NSTEMI* non-ST elevation myocardial infarction; *SBP* systolic blood pressure; *STEMI* ST elevation myocardial infarction; *UA* unstable angina

**Fig. 2:**
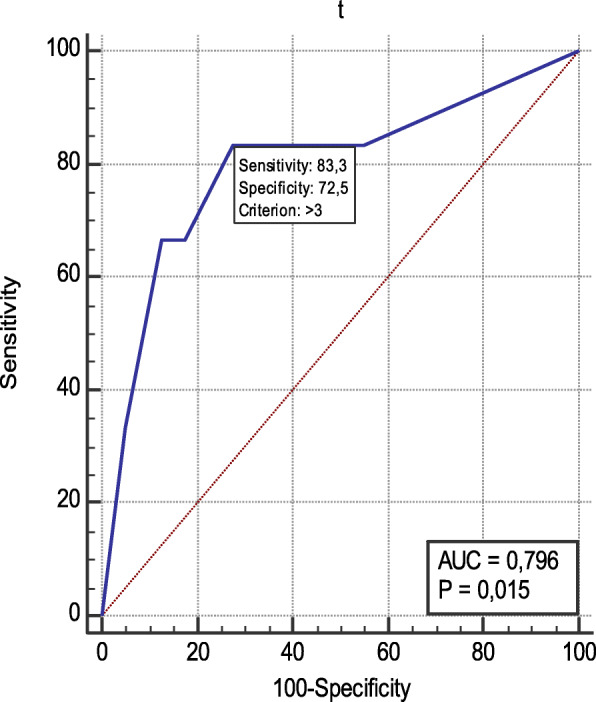
Showing sensitivity and specificity of no. of leads with f-QRS to in-hospital death

The ROC curve showed that the optimal cutoff value of f-QRS leads is > 3 f-QRS leads for predicting hospital mortality with 83.3% sensitivity and 72.5% specificity. Area under the curve AUC = 0.796; 95% CI = 0.651 to 0.900 (*P* = 0.015) (Fig. 2).

- *Impact of number and location of f-QRS*. The fragmented QRS cases group was further subdivided into 2 subgroups according to the numbers of f-QRS leads. Subgroup (A1) included patients with four f-QRS leads or more and subgroup (A2) included patients with 3 or fewer f-QRS leads. Subgroup (A1) showed statistically significant difference lower SBP (111.33 ± 25.03 mmHg vs. 139 ± 38.89 mmHg, *P* = 0.016), a higher HR (93.81 ± 19.13beats/min vs. 80.77 ± 14.91beats/min, *P* = 0.014); additionally higher updated GRACE risk score (6.81 ± 12 vs. 3.22 ± 6.95, *P* = 0.048) compared to subgroup A2. Subgroup A1 also showed significantly a higher number of in-hospital mortality (5/16 vs. 1/30, *P* = 0.015) than subgroup A2 (Tables 2 and 3).

**Table 2:** Echocardiography data

	f-QRS	Non f-QRS	***P*** value
**LVEDD,** mm	52.98 ± 7.097	49.44 ± 6.89	*0.033*
**LVEF,** (%)	53.78 ± 12.016	55.67 ± 11.962	*0.493*
**No systolic dysfunction**	28/41	25/36	*0.913*
**Mild systolic dysfunction**	9/41	8/36	*0.977*
**Moderate systolic dysfunction**	2/41	3/36	*0.660*
**Severe systolic dysfunction**	2/41	0/36	*0.496*
**No diastolic dysfunction**	0/39	1/32	*0.451*
**Diastolic dysfunction I**	28/39	19/32	*0.271*
**Diastolic dysfunction II**	9/39	10/32	*0.439*
**Diastolic dysfunction III**	2/39	2/32	*1.000*
**No MR**	22/41	22/35	*0.418*
**Mild MR**	14/41	6/35	*0.093*
**Moderate MR**	4/41	7/35	*0.327*
**Severe MR**	1/41	0/35	*1.000*
**In-hospital death**	6/46	5/38	*1.000*

*LVEDD* left ventricle end diastolic diameter; *MR* mitral regurgitation; *PCI* percutaneous coronary intervention; *RCA* right coronary artery; *SBP* systolic blood pressure

**Table 3:** The important results of the 2 subgroups (A1) and (A2)

	Subgroup (A1) > 3fQRS	Subgroup (A2) ≤ 3f-QRS	***P*** value
**SBP,** mmHg	111.33 ± 25.03	139 ± 38.89	*0.016*
**Heart rate,** bpm	93.81 ± 19.13	80.77 ± 14.91	*0.014*
**Abnormal cardiac biomarkers**	13/16	28/30	*0.325*
**Killip (IV)**	3/16	1/30	*0.114*
**In-hospital death**	5/16	1/30	*0.015*
**Updated GRACE score**	6.81 ± 12	3.22 ± 6.95	*0.048*
**LVEF (%)**	48.08 ± 13.07	56.14 ± 10.92	*0.049*

*LVEF* left ventricle ejection fraction; *SBP* systolic blood pressure

**Fig. 3:**
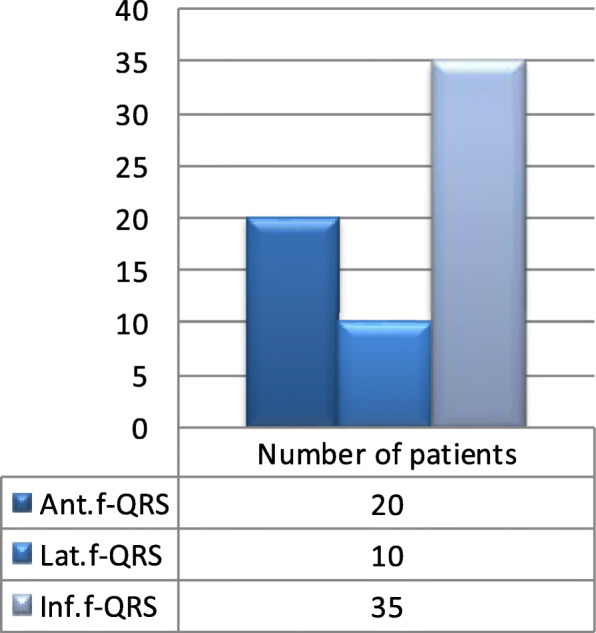
Frequency of distribution of location of f-QRS lead

Anterior location of f-QRS was present in 20 out of 46 patients (43.5 %) of the cases group. While inferior location of f-QRS was encountered in 76.1% of patients while the lateral location of f-QRS was the least (21.7%) in the patients studied (Fig. 3). The different fragmented QRS locations can have variable impacts on hemodynamics, the occurrence of cardiogenic shock and updated GRACE risk score, and in-hospital mortality. The Anterior f-QRS location showed significant statistical lower SBP, a higher HR, a higher incidence of cardiogenic shock (KILLIP class IV), a higher updated GRACE risk score (*P* = 0.033), and a higher incidence of in-hospital mortality (*P* = 0.004) compared to non-anterior fragmented-QRS location (Table 4).

**Table 4:** Relations between anterior f-QRS and some important medical results in the cases group

	Anterior f-QRS	Non-anterior f-QRS	***P*** value
**SBP,** mmHg	112.6 ± 23.5	142.31 ± 40.33	*0.006*
**HR,** bpm	91.3 ± 18.62	80.7 ± 15.3	*0.040*
**Abnormal cardiac biomarkers**	17/20	24/26	*0.64*
**Killip (IV)**	4/20	0/26	*0.030*
**Updated GRACE score**	7.68 ± 13.17	2.03 ± 1.72	*0.033*
**LVEF (%)**	48.7 ± 12.03	56.7 ± 11.22	*0.039*
**In-hospital death**	6/20	0/26	*0.004*

*HR* heart rate; *LVEF* left ventricle ejection fraction; *SBP* systolic blood pressure

## Discussion

[8] acknowledged that despite numerous studies, the predictive value of f-QRS in the 12-lead ECG in patients after acute coronary syndrome is controversial. Research carried out in large groups of patients provides conflicting results, therefore, there is a need to carry out a number of meta-analysis in order to draw the right conclusions.

In the present study, we meant to detect whether the f-QRS presence has any role in the prediction of poor outcomes in patients with acute coronary syndrome. During the study, we have found that it is not the mere presence of f-QRS that affect the outcomes, but its location and the number of fragmented leads that matter.

In our study, f-QRS presented in 46 out of 84 patients (54.8%) with acute coronary syndrome. These 2 groups did not present any significant difference regarding general demographic characteristics and the most important risk factors of coronary heart disease. These results matched those in [3] by Bekler and associates. On examination of the patients enrolled in the study, measurement of SBP and HR as vital signs showed no significant difference between both groups.

[8] compared HR between patients with acute coronary syndrome according to the occurrence of f-QRS using Holter, average of HR between both groups was not statistically significant (*P* value was 0.33).

There was no significant relation between both main groups regarding positive cardiac bio-markers(*P* = 0.534), in the study conducted by [1], *P* value of elevated CPK and troponin I between f-QRS group and non f-QRS group was 0.39 and 0.60 respectively.

We assessed the Killip class between both groups with more focus on Killip IV which represents cardiogenic shock. There were no significant differences between them, [10] defined heart failure as a Killip class ˃ 1, there was no significant difference between the three groups of the study (persistent f-QRS, transient f-QRS and non f-QRS) as regards Killip class ˃ 1.

The updated GRACE risk score was higher in group (A) with no significant difference between both groups (*P* = 0.899). However, [4] succeeded to find a higher and significant GRACE score in group with f-QRS than non f-QRS group, this dissimilarity in both studies results is believed to be due to different patients populations as they excluded STEMI and UA from their study, our study included all variants of acute coronary syndrome.

In the term of in-hospital death, there was not any significant difference between the both groups (*P* = > 0.99). In NSTEMI, there was no significant difference between f-QRS group and non f-QRS regarding mortality in the study conducted by [11]. From the above information, we can find that presence of f-QRS in ECG of patients with ACS does not add any significant prognostic value (Table 1), but the impact of number or location of fragmented leads has great value as seen next.

Receiver operator characteristic (ROC) analysis showed that greater than 3fragmented QRS was the optimum cutoff value for number of f-QRS leads in predicting in-hospital mortality (Fig. 2), these findings are almost in agreement with the study conducted by [14] where it concluded that the presence of ≥ 3 f-QRS leads is independently connected with cardiac mortality or hospitalization for heart failure in 170 patients with past MI .Thereby, we divided the cases group into 2 subgroups according to the numbers of f-QRS leads. Subgroup (A1) included patients with more than 3 f-QRS leads and subgroup (A2) included patients with 3 or less f-QRS leads. Moreover, the case group was also divided according to the location of f-QRS into three groups (anterior, lateral, and inferior). Subgroup (A1) showed a significant difference in the term of SBP and HR readings on admission where it showed lower and higher values respectively than subgroup (A2) (Tables 2 and 3). Anterior f-QRS was also associated with significant higher HR and lower SBP than non-anterior f-QRS (Table 4). Number of f-QRS was not significantly related to Killip IV and both subgroups did not show any significant difference (*P* = 0.114). Only anterior f-QRS when compared to non-anterior f-QRS showed the significant difference (*P* = 0.030). Patients in the subgroup (A1) had a higher and significant updated GRACE risk score than subgroup (A2). Furthermore, anterior location of f-QRS had statistically significant higher updated GRACE risk score (*P* = 0.033) than non-anterior f-QRS.

Finally, in terms of in-hospital death, subgroup (A1) showed a significantly higher incidence of in-hospital death in relation to subgroup (A2) (*P* = 0.015) [12]., in their research on the value of the number of fragmented QRS leads in the prediction of in-hospital mortality in acute STEMI patients treated with primary PCI. They concluded that the number of f-QRS leads was significantly higher among patients with in-hospital mortality.

Anterior f-QRS location was associated significantly with in-hospital death. Terho and colleagues concluded in [13], that f-QRS in lateral leads in patients with confirmed cardiac diseases was associated with higher risk of all-cause death, it is worth noting that they considered lateral leads territory on ECG was (I, aVL, V4 to V6) while in our study we considered lateral f-QRS territory only in ( I, aVL, and V6) and anterior leads ( V1 to V5) as [10, 15] have done. Based on the above analysis, our explanation to the non-significant result between the two major groups is thought to be due to that f-QRS in 2 and 3 leads presented in 39% and 26% of the fragmented group respectively.

### Study limitations

Study limitations include the relatively small sample, retrospective with less precisive and available data, no inclusion of f-QRS in aVR, V3r, and V4r leads, no study of arrhythmia, re-infarction or long term mortality, and finally, the low pass filter that is optimally used in order to detect f-QRS is 100-150 Hz, it may be masked when using filter with a lower setting.

## Conclusion

F-QRS in > 3 leads and anterior f-QRS have significant lower systolic blood pressure and heart rate and significant higher heart rate, updated GRACE risk score and incidence of in-hospital mortality. Moreover, anterior f-QRS is associated with higher incidence of Killip IV (cardiogenic shock).

## Recommendation

The mere presence of f-QRS on 12 leads surface ECG in patients with ACS is not enough to predict the prognosis; however, using the number if greater than 3f-QRS leads and the anterior location of f-QRS leads is of a great value to predict the prognosis, the occurrence of cardiogenic shock and in-hospital mortality (Fig. 3).

## Data Availability

Data and material are available upon request.

## Abbreviations

ACS: Acute coronary syndrome
AF: Atrial fibrillation
BBB: Bundle branch block
CABG: Coronary artery bypass graft
CAD: Coronary artery diseases
CHD: Coronary heart disease
DM: Diabetes mellitus
ECG: Electrocardiogram
EF: Ejection fraction
f-QR: Fragmented QRS
GRACE: Global Registry of Acute Coronary Events
HR: Heart rate
HTN: Hypertension
Hz: Hertz
MACE: Major advanced cardiac events
MI: Myocardial infarction
NSTEMI: Non-ST elevation myocardial infarction
PCI: Percutaneous coronary intervention
ROC: Receiver operator characteristic
SBP: Systolic blood pressure
SD: Standard deviation
STEMI: ST elevation myocardial infarction
UA: Unstable angina
